# Dose-efficient scanning Compton X-ray microscopy

**DOI:** 10.1038/s41377-023-01176-5

**Published:** 2023-05-30

**Authors:** Tang Li, J. Lukas Dresselhaus, Nikolay Ivanov, Mauro Prasciolu, Holger Fleckenstein, Oleksandr Yefanov, Wenhui Zhang, David Pennicard, Ann-Christin Dippel, Olof Gutowski, Pablo Villanueva-Perez, Henry N. Chapman, Saša Bajt

**Affiliations:** 1 The Hamburg Centre for Ultrafast Imaging, 22761 Hamburg, Germany; 2 Center for Free-Electron Laser Science CFEL, Deutsches Elektronen-Synchrotron DESY, 22607 Hamburg, Germany; 3 Deutsches Elektronen Synchrotron DESY, 22607 Hamburg, Germany; 4 Synchrotron Radiation Research and NanoLund, Lund University, 221 00 Lund, Sweden; 5 Department of Physics, Universität Hamburg, 22761 Hamburg, Germany

**Keywords:** Optical techniques, Microscopy

## Abstract

The highest resolution of images of soft matter and biological materials is ultimately limited by modification of the structure, induced by the necessarily high energy of short-wavelength radiation. Imaging the inelastically scattered X-rays at a photon energy of 60 keV (0.02 nm wavelength) offers greater signal per energy transferred to the sample than coherent-scattering techniques such as phase-contrast microscopy and projection holography. We present images of dried, unstained, and unfixed biological objects obtained by scanning Compton X-ray microscopy, at a resolution of about 70 nm. This microscope was realised using novel wedged multilayer Laue lenses that were fabricated to sub-ångström precision, a new wavefront measurement scheme for hard X rays, and efficient pixel-array detectors. The doses required to form these images were as little as 0.02% of the tolerable dose and 0.05% of that needed for phase-contrast imaging at similar resolution using 17 keV photon energy. The images obtained provide a quantitative map of the projected mass density in the sample, as confirmed by imaging a silicon wedge. Based on these results, we find that it should be possible to obtain radiation damage-free images of biological samples at a resolution below 10 nm.

## Abstract (web summary)

High-resolution, low-dose images of biological objects were obtained in a new method using photons of 60 keV. The dose was thousands of times lower than conventional methods, overcoming the previous limitations set by radiation damage to the sample.

## Graphical abstract

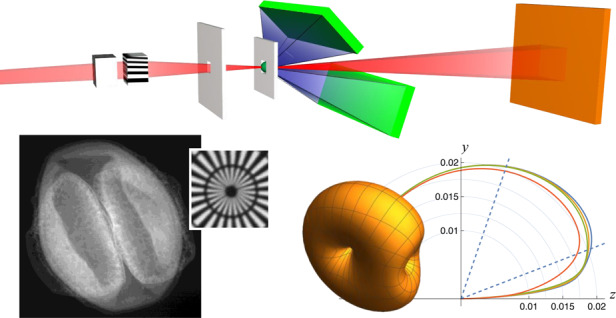

## Introduction

Short wavelength radiation, such as X-rays or electrons, can in principle be used to form images at a spatial resolution that approaches the atomic scale. However, radiation at ångström wavelengths is energetic enough to ionise the material under study, initiating processes that cause structural changes that limit the quality of the image of the sample. This is particularly acute for biological materials, which can usually only withstand a certain maximum dose before this radiation damage of the structure is observable at a particular resolution^1^. For cryogenically cooled hydrated biological samples, the length scale of the structural degradation scales approximately linearly with the dose. Thus, the tolerable dose scales with the resolution and is found to be about 100 MGy for each 1 nm of degradation^1^. Imaging at 10 nm resolution must therefore be accomplished while imparting a dose of no more than 1 GGy. This condition is readily satisfied with 200 keV electrons to produce images of biological samples at 4 nm resolution at an exposure of about 1.2 e/Å^2^ (ref. ^2^), but such imaging is limited by multiple scattering to samples thinner than about 0.6 µm. Soft X-rays, operating in the “water window” of photon energies between 0.28 keV and 0.53 keV, can produce dose-limited images of cells at 25 nm resolution or better, but may be limited by the depth of focus of optics required at these relatively long wavelengths^3^. Phase-contrast X-ray imaging^4^, including ptychography^5^ and projection holography^6^, at around 10 keV photon energy have proven useful since this radiation is more penetrating through the sample and the shorter wavelengths give larger depths of focus. These wavelengths also provide longer working distances of diffractive lenses, an advantage for performing tomographic imaging. Recently, the use of even higher energy photons at 60 keV was investigated for a low-dose scanning microscopy based on Compton scattering^7,8^. In this approach, maps of the number of detected incoherently scattered photons as a function of the position of the focused beam provide images of the electron density of the sample. Fluorescence counts and elastic scattering (coherent diffraction) may also contribute to the image, or be discriminated from the Compton signal to produce multi-spectral images. Our first theoretical and experimental investigations suggested that, depending on sample size, scanning Compton X-ray microscopy could provide high-resolution images at lower dose than phase-contrast imaging. Here we report on tests of this method using a 60 keV probe beam focused to about 70 nm using multilayer Laue lenses. We show the applicability to imaging biological objects and present images of dried objects—a spirulina bacterium, a diatom, and a pollen grain—acquired at doses far below tolerable limits for cooled hydrated samples. Given that the dose to achieve a particular signal to noise ratio in incoherent imaging generally scales with the inverse fourth power of the resolution length^7^ and that the tolerable dose increases linearly with the resolution length^1^, we predict a damage-limited resolution of about 9 nm for the pollen grain and diatom, and 15 nm for the bacterium. Achieving a practical implementation of high-resolution scanning Compton X-ray microscopy relies upon the development of effective detectors with large solid angle^9^ and the increased brightness of next-generation synchrotron radiation sources^10^.

X-rays interact with matter in a variety of ways, and microscopy can be generalised as the localisation of particular interactions by detecting the presence or absence of emanating radiation^11^. Low-dose imaging requires maximising the detectable signals for a given amount of energy transferred to the sample. In the water window this is achieved by measuring the attenuation of the beam, where the signal is directly proportional to the energy deposited into the sample. With increasing photon energy, the optimum signal per energy deposition shifts to elastic scattering as both the absorption and elastic scattering cross sections decrease, and then to inelastic (Compton) scattering as this interaction becomes dominant. For samples consisting of light elements the least dose per signal is achieved at about 50 to 70 keV, beyond which the photon energy loss per inelastic event is a significant cost for the information that it yields^7^. The lack of focusing optics for high photon energies has limited the achievable resolution in Compton scattering-based imaging techniques. So-called Compton scattering imaging or backscatter methods have therefore largely been developed to image large objects where high penetration is needed (such as airport security screening^12^) and where the detection in the backscattering geometry is advantageous (such as investigating historical artefacts^13^). Scanned-beam Compton imaging has been performed using a pencil-beam of synchrotron radiation, formed by a pinhole, to image lithium in an operating battery^14^. Recently, a light-sheet approach has been adopted for battery research, where a pinhole image is formed of the Compton-scattered radiation perpendicular to the illuminating sheet^15^, and it has been suggested to improve imaging speed by replacing the pinhole with a coded mask^16^. Here, we carry out scanned-beam scanning Compton X-ray microscopy at unprecedented spatial resolution with novel thick, wedged multilayer Laue lenses developed specifically for focusing high-energy X-rays. We particularly aim to maximise the detected signal in order to reduce the imaging dose.

## Results

**Fig. 1:**
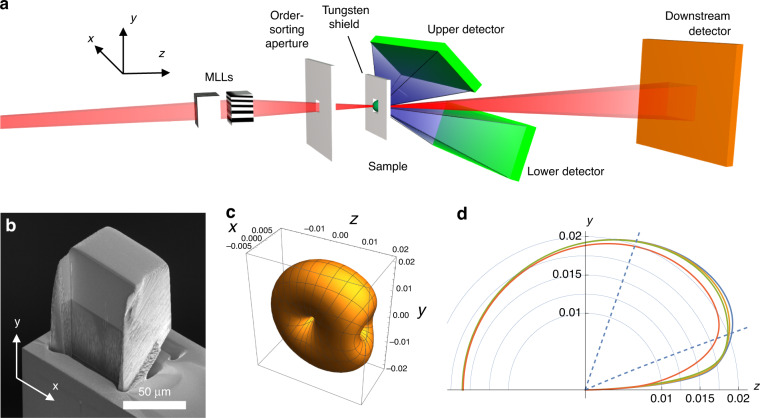
Scanning Compton X-ray microscopy. **a** Schematic of the microscope. **b** Scanning electron micrograph of one of the multilayer Laue lenses, 35 µm thick in the beam propagation direction (*z*) and 51 µm high (*y*). The multilayer structure has a grey colour above the Si substrate, which appears darker. **c** Differential cross section of carbon in units of cm^2^/g/solid angle at 60 keV photon energy, and **d** slices of the differential cross sections in the same units of C (blue), N (yellow), O (green), and Si (orange). The acceptance of the upper detector is indicated by the dashed lines

We demonstrated scanning Compton X-ray microscopy using 60 keV photons (0.02 nm wavelength) at beamline P07 of the PETRA III synchrotron radiation facility with the setup shown schematically in Fig. 1a. The beam was focused onto the sample with a pair of wedged multilayer Laue lenses^17,18^. These diffractive optics consist of alternating layers of SiC and WC which diminish in period with distance from the optical axis. X-rays are diffracted by increasing angles with this distance so as to converge at a common point, the focus. Two lenses, oriented orthogonally, are used to focus in both directions. The resolution of that focus is governed by the numerical aperture (NA) of the lens, determined by the range of diffraction angles. Given the inverse relationships between period and diffraction angle, and NA and resolution, the focal spot size is proportional to the smallest period in the diffracting structure—which was 4.4 nm in this case. A challenge is the low coherent scattering cross section of materials at 60 keV. To counteract this, the lens structures must have a thickness in the beam propagation direction of about 35 µm to ensure that intensity is transferred into the diffracted (i.e. focused) beam, but the low absorption nevertheless guarantees high diffraction efficiency. The layers in the lenses consequently must have an aspect ratio (period to length) approaching 1/10 000, which was achieved here by layer deposition by magnetron sputtering followed by slicing (see Methods). Each lens is thicker than the depth of focus, and the layer aspect ratio is smaller than the lens NA. The layers must therefore be accurately tilted and wedged to ensure the Bragg condition is satisfied for all layers^19^ to create waves that constructively interfere at the focus^20^. One lens is shown in Fig. 1b, with a height of about 51 µm and focal length of 15.6 mm. The other lens had a 15.0 mm focal length and placed 0.6 mm downstream to focus to a common plane.

A 3D plot of the differential scattering cross section per unit mass of carbon at 60 keV is given in Fig. 1c for an incident beam that is polarised in the horizontal (*x-*z) plane. The Compton scattering cross section per atom at high photon energies varies approximately with the number of electrons, *Z* (the atomic number)^21^, and thus the cross section per unit mass of different species follows *Z⁄A*_*Z*_ where *A*_*Z*_ is the atomic mass. Except for hydrogen, this ratio is fairly similar for different light elements as can be seen in Fig. 1d. Although the highest possible signal would be obtained by measuring scattering over the complete 4π solid angle (or as close to it as possible^9^), in these measurements we used two pixel-array photon-counting detectors placed close to the sample, above and below the optical axis, capturing scattering angles between 23° and 80° for the upper detector and 5° to 30° for the lower—see Fig. 1a. The detector threshold energy for photon counting was set to 20 keV. Both of the detectors had 1536 × 512 square pixels of 55 µm width, and they were positioned with the long edge parallel to the *x* axis (along which the scattering decreases most rapidly with angle) rather than the short edge. Due to the shape of their housings, this orientation allowed the detectors to be placed closer to the sample to maximise the solid angle and recorded scattering signal. However, the positioning of the lower detector was constrained by other instrumentation and was oriented at a shallower scattering angle. Since the attenuation of 60 keV photons is negligible in soft-matter samples that are considerably thinner than 1 mm, the measured scattering signal scales with the density and thickness of each material in the beam and the measured signal can be given as

1 $$N={I}_{0}\varDelta t\sum _{Z}{\sigma }_{Z}^{{\rm{det }}}{\rho }_{Z}{t}_{Z}$$

where *I*_0_ is the incident photon flux, *Δt* the exposure time, *ρ*_*Z*_ and *t*_*Z*_ are the partial densities and thicknesses of the atomic species in the sample (e.g. carbon), and ${\sigma }_{Z}^{{\rm{det }}}$ is the integral of the differential cross section per unit mass of that element over the solid angle of the detector and accounting for the detector quantum efficiency. As indicated by Fig. 1d, for light elements, the sum in Eq. (1) can be well approximated by the projected density of the sample, *ρt*, multiplied by an average detector-integrated cross section for those elements.

A map of the detected signal *N* as a function of position of the object as it is raster scanned in the plane transverse to the beam thus gives an image of the projected electron density. This is a dark-field image, in that when there is no sample in the beam there should be no signal. In practice this ideal is hard to achieve due to scattering from the air (which could be considered to be part of the sample) and scattering from the optics and apertures in the beam. This background was minimised by mounting the sample in a 1-mm hole in a tungsten plate that blocked scattered light from upstream components, and by attaching a pyramid-shaped Pb shield to each of the off-axis detectors. Each pyramid tip was truncated to leave a hole about 5 mm wide, close to the sample. The sensors of the two off-axis detectors were CdTe which has 95% efficiency at 60 keV. A third, unshielded, pixel-array detector with a GaAs sensor was placed 4.2 m downstream of the sample and used to align and characterise the lenses, as described below, and to measure the transmitted beam. The air path and a 15 mm thick Si crystal attenuator reduced the 62% quantum efficiency of this detector to 18%.

**Fig. 2:**
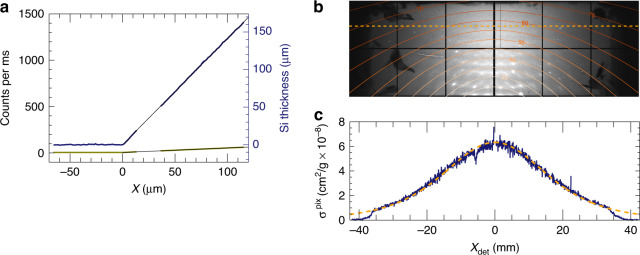
Calibration of the Compton scattering signal. **a** Measured signals in the upper (blue) and lower (green) off-axis detectors from a silicon wedge sample, made with 2000 ms exposures at each point and an incident flux of 1.54 × 10^6^ ph/ms. **b** Background-subtracted detector per-pixel cross section of Si, shown on a greyscale ranging from 0 (black) to 8 × 10^−8^ cm^2^/g (white). Contours of the scattering angle *θ* are indicated. **c** A line-out of the response along the dashed orange line of (**b**), and the corresponding computed cross section (dashed line)

To determine *σ*^det^ for the two dark-field detectors, we first imaged a wedge of crystalline silicon made by anisotropic etching of a [100] wafer to expose a face normal to [111]. The thickness *t* of the wedge at a scan position *x* from the vertex along the [110] direction (perpendicular to the edge) is $t=\sqrt{2}x$. Plots of the signals in the two detectors are shown in Fig. 2a, together with straight-line fits which provide a scaling of 8.11 ph/ms/µm of Si for the upper detector compared with only 0.35 ph/ms/µm for the lower detector. Away from the Si wedge, at positions *x* < 0, a background signal of 88.1 ph/ms was observed in the upper detector and 5.55 ph/ms in the lower. These background signals ultimately limit the smallest variations in projected density that can be measured, as discussed below, and were further reduced in subsequent measurements. Given the incident counts of 1.54 × 10^6^ ph/ms, as measured with the downstream detector, and the known density of Si, 2.329 g/cm^3^, the slopes of the lines in Fig. 2a yield the cross sections of ${\sigma }_{{\rm{Si}}}^{u}=$ 0.0227 cm^2^/g for the upper detector and ${\sigma }_{{\rm{Si}}}^{d}=$ 0.0010 cm^2^/g for the lower. These can be compared with the total Compton scattering cross section of Si at 60 keV (into 4π steradian), equal to 0.153 cm^2^/g (ref. ^22^).

The off-axis detectors collect elastic scattering (and potentially fluorescence photons) in addition to the Compton signal. All these signals contribute to the image and to the dose efficiency of the method. Both the total elastic scattering and photoabsorption cross sections are approximately proportional to *Z*^2^, although heavier elements direct more of the elastic scattering towards the forward direction. A map of the signal recorded in the upper detector from Si is shown in Fig. 2b after subtracting the background (no sample) signal. Besides the Si Bragg peaks, the highest diffuse intensity is seen along the axis at *x*_det_ = 0 due to the distribution of the differential cross section for Compton scattering (see Fig. 1c) as well as the larger solid angle of pixels along that axis. Some shadows occur near the edges of the detector due to the lead shielding that was used, seen as dark regions in Fig. 2b. The diffuse elastic scattering of disordered materials can be calculated from the atomic scattering factors, and contribute 5% of the total signal in the upper detector. Clearly, Si is not disordered—the Bragg peaks extend to a scattering angle of *θ* = 45° (0.26 Å resolution). The residual signal after subtracting the calculated Compton signal and disordered elastic signal accounts for about 2% of the total (see Methods). A line out from the background-subtracted signal is also shown in Fig. 2c, converted into single-pixel Compton scattering cross sections per unit mass and compared with the calculation. A high agreement is found, achieved by adjusting the sample to detector distance in the calculation to 31.0 mm (compared with our nominal setting of 30 mm). Organic objects consisting primarily of C, N, and O (by mass) will give similar total cross sections but with a lower proportion of elastic scattering. For example, the upper-detector cross sections for N are calculated to be 0.023 cm^2^/g (Compton) + 0.001 cm^2^/g (elastic). As the atomic number *Z* increases, the proportion of the signal caused by elastic scattering increases slightly but at 60 keV photon energy and with our detector configuration the Compton signal dominates for all elements. However, for elements heavier than Si, the photoabsorption cross section at 60 keV is greater than the total Compton scattering cross section, giving rise to fluorescence as the dominant signal (depending on the element and the spectral response of the detector).

**Fig. 3:**
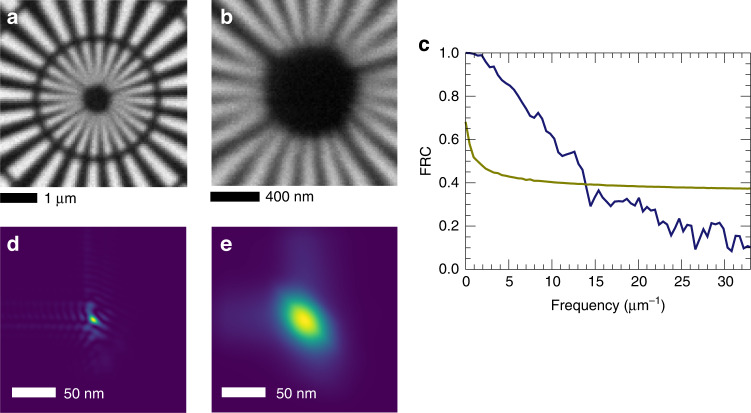
Imaging resolution. **a**, **b** Dark-field images of a 750-nm thick Siemens star structure measured with step sizes of 50 nm and 15 nm, and dwell times per pixel of 10 ms and 20 ms, respectively. **c** Fourier ring correlation formed from the scan of (**b**) and an identical scan, indicating a resolution of 72 nm. The one-bit criterion is shown in olive green. **d** The point spread function of the lenses, obtained from a measurement of the wavefront. **e** The calculated focal spot for a source of 100 µm x 100 µm at a distance of 42 m and a relative bandwidth of 0.05%

To characterise the achievable resolution of dark-field images, scanned images were obtained from a 750-nm thick gold Siemens star structure, as shown in Fig. 3. The Fourier ring correlation (FRC) of two separate images of this object gives a measure of this resolution as 72 nm according to the one-bit criterion^23^ (Fig. 3c). Dark-field images formed this way are incoherent images, given by the convolution of the intensity profile of the focused probe with the projected electron density^24^. Nevertheless, the smallest possible spot size requires both low enough lens aberrations and a high degree of coherence of the illuminating beam achieved with a source of small enough angular extent. The wavefront aberration of the lens pair at 60 keV photon energy was measured by the method of ptychographic speckle tracking^25^, using magnified projection images of a gold Siemens star object measured on the downstream pixel-array detector located 4.2 m from the sample. The high density and high atomic number of gold ensures that the Siemens star object causes a small but measurable perturbation to the transmitted wavefront, as needed for wavefront sensing via the speckle tracking technique (see Methods). A fully coherent beam would produce a point spread function (PSF) obtained from this wavefront that measures 7.6 nm × 7.6 nm as shown in Fig. 3d. The limited source brightness at 60 keV, even at a storage-ring as large as PETRA III, requires a compromise between flux and effective source size. At the P07 beamline, the source was the exit aperture of a Si crystal monochromator, located 30 m upstream of the lenses. This was set to a size of 100 µm × 100 µm, producing a relative bandwidth of about 0.05% and broadening the PSF to a width less than the geometrical image of 50 nm. The relative bandwidth is larger than the inverse of the number of periods in the lens, causing a chromatic aberration. Since the apertures of both lenses are displaced from the optical axis, this aberration leads to a diagonal broadening of the focused spot, depicted in Fig. 3e, with a width of about 55 nm in this direction. The flux through the focal spot was 1.56 × 10^6^ photons/ms as measured on the downstream detector.

### Compton images of biological objects

**Fig. 4:**
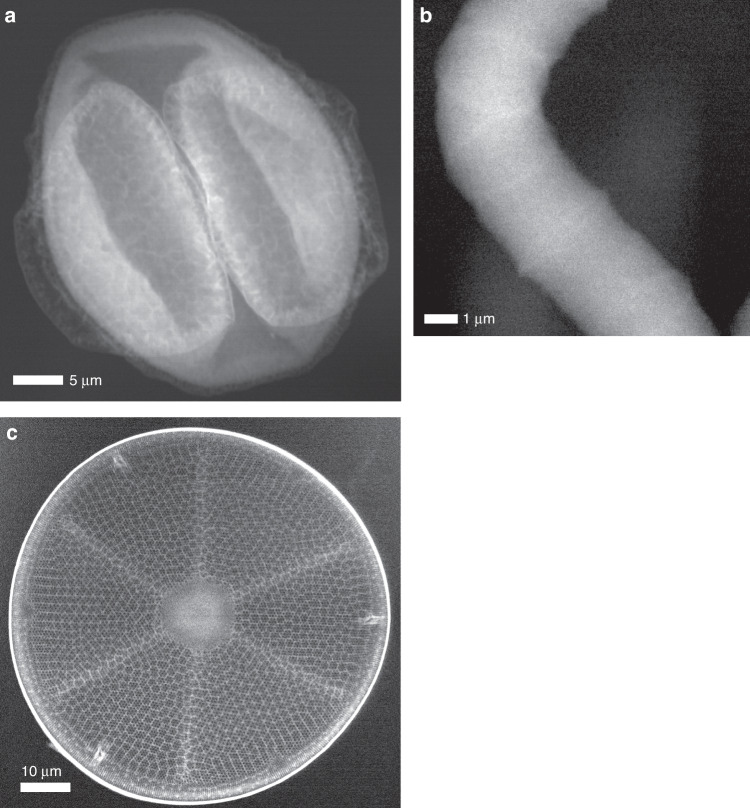
Dark-field images. **a** A pine pollen grain, (**b**) a spirulina blue-green algae, and (**c**) a silicate diatom shell. The scale bars are indicated, and the parameters for the scans are given in Table 1. The colour scale ranges from 0 (black) to the maximum (white) projected density given in Table 1

Dark-field images of three biological objects are shown in Fig. 4—a pine pollen grain, a spirulina cyanobacterium, and a diatom shell. All three samples were dry, unstained, and unfixed. The samples were separately mounted on silicon nitride membranes 100 nm thick. Parameters of the scans and the displayed images are given in Table 1. The resolution of these images is limited by the step sizes, so they do not display the achievable resolution obtained for the gold structure. The images are quantitative, and the grey-scale is proportional to the projected density, computed assuming the detector cross section for the stoichiometries listed in Table 1, although the choice of stoichiometry changes the result by less than 1%. White represents higher projected density. Since the densities of these objects are slightly greater than 1 g/cm^3^, the quantities are close to the thickness, in micrometers. The diatom and pollen grain show large variations in projected density due to the porous structure of the diatom shell and the nanofoam structure of the pollen wall called the exine^26^. The cavities in this foam range in diameter from about 0.5 µm to 2 µm and the thickness of the exine, apparent around the circumference of the projected image, is about 1 µm, consistent with X-ray phase-contrast tomography measurements of pine pollen^26^. The spirulina has a structure of a helical wire of about 4 µm wire diameter with a coil pitch of 20 µm and coil diameter of about 10 µm. The image of the spirulina organism (Fig. 4b) does not show strong contrast although faint cell walls, of higher density, can be discerned which cross the width of the structure. The maximum projected density of the spirulina object is 5.7 g/cm^3^ µm, compared to the width of the object in the plane of the image of 3.7 µm. That is, the thickness would be roughly equal the width for a density of 1.5 g/cm^3^. The lack of discernible structures in the spirulina cells is consistent with published optical and transmission electron microscope images^27^.

**Table 1:** Parameters of dark-field images displayed in Fig. 4, cross sections for photoabsorption and inelastic energy loss, and dose, given the stoichiometries listed (see text and Eq. 1 and 2)

	Pine pollen grain	Spirulina	Silicate diatom
Incident intensity (ph/ms)	1.63e+06	1.7e+06	1.50e+06
Exposure dwell time (ms)	50	150	24
Step size (nm)	50	30	60
Total exposure time	8 h 54 m	6 h 40 m	9 h 54 m
Assumed composition	H_36_C_26_NO_18_	H_50_C_30_N_9_O_10_S	SiO_2_
*σ*^det^ (cm^2^/g)	0.025	0.025	0.027
*σ*_*a*_ (cm^2^/g)	0.010	0.017	0.051
${\bar{{{\sigma }}}}_{{{inc}}}$ (cm^2^/g)	0.016	0.016	0.015
Dose (MGy)	0.81	9.0	0.63
Minimum (black) counts / ms	42	93	40
Maximum (white) counts / ms	173	135	114
Projected density (g/cm3 µm)	33.7	5.7	2.1

The projected density is that corresponding to the full scale (black to white) of the displayed image

The calculated radiation dose imparted to each sample in creating the images of Fig. 4 is given in Table 1. Energy from the X-ray beam is imparted both through photoabsorption, in which the entire photon energy is transferred to the sample, and the inelastic process of Compton scattering, where a fraction of the photon energy is transferred, dependent on the scattering angle. Since the integration over all scattering angles is independent of polarisation the dose is calculated as^7^

2a $$D=\frac{{E}_{{\rm{in}}}{I}_{o}\varDelta t}{{A}_{0}}\left({\sigma }_{a}+{\bar{\sigma }}_{{\rm{inc}}}\left({E}_{{\rm{in}}}\right)\right)$$

2b $${\bar{\sigma }}_{{\rm{inc}}}\left({E}_{{\rm{in}}}\right)=\int \frac{d{\sigma }_{{\rm{inc}}}}{d\Omega }\left(1-p\left({E}_{{\rm{in}}},\theta \right)\right)d\Omega$$

where

3 $$p\left({E}_{{\rm{in}}},\theta \right)=\frac{{E}_{{\rm{out}}}}{{E}_{{\rm{in}}}}=\frac{1}{1+\frac{{E}_{{\rm{in}}}}{{m}_{e}{c}^{2}}(1-{\rm{cos }}\,\theta )}$$

is the ratio of the scattered photon energy *E*_out_ to the incident photon energy *E*_in_ and dependent on *m*_*e*_*c*^2^ = 511 keV, so that *E*_in_(1-*p*) is the energy transferred to the matter in the interaction. *A*_0_ is the exposed area, *σ*_*a*_ is the total photoabsorption cross section per unit mass, and ${\bar{\sigma }}_{{\rm{inc}}}$ is the energy-loss cross section for inelastic scattering. As given by Eq. (2a), this cross section is weighted by the fraction of energy transferred into the sample. Values of the contributions *σ*_*a*_ and ${\bar{\sigma }}_{{\rm{inc}}}$ to the dose are given in Table 1 for the three samples. These were computed for the composition of pine pollen given by Filipiak^28^ and an average protein composition was used for spirulina^1^. As seen in the Table, the inelastic cross sections per mass are not sensitive to the stoichiometry for light elements. The doses to the pollen grain and spirulina were roughly equally partitioned to photoabsorption and inelastic energy loss, whereas photoabsorption was the dominant process for the silicate diatom. This ratio can be tuned by changing the incident photon energy. Increasing the photon energy *E*_in_ beyond 60 keV reduces photoabsorption faster than the Compton cross section, but each photon interaction imparts more energy. For the pollen and spirulina, the signal per dose, proportional to ${\sigma }^{{\rm{det }}}/({E}_{{\rm{in}}}\left({\sigma }_{a}+{\bar{\sigma }}_{{\rm{inc}}}\right))$, is a maximum at 60 keV. The diatom, consisting of slightly heavier elements, can be imaged with 30% more signal for a given dose at an optimal photon energy of 95 keV.

## Discussion

The doses to the samples are far below the tolerable levels for imaging unfixed biological objects. At a resolution of 70 nm, this tolerable dose is ~7 GGy (ref. ^1^), allowing exposures >8000 times stronger in the case of the pollen. Although it was not feasible for us to carry out phase-contrast X-ray imaging experiments on the same or similar samples, recent published results of holographic tomography of pine pollen grains^26^ do offer this comparison. That work was carried out at the European Synchrotron Radiation Source (ESRF) using a photon energy of 17 keV, and yielded a resolution in all three dimensions similar to the two-dimensional resolution achieved here. The chosen photon energy gave close to the maximum phase shift per absorption, as determined by the ratio of the real to imaginary parts of the refractive index of soft matter^29^. Even so, it is notable that the exposures were obtained with a photon exposure *I*_0_*Δt* that was 100 times greater than used here and samples were cryogenically cooled as a precaution against effects of radiation damage. The dose is estimated as 1.8 GGy, assuming the same composition as given in Table 1. This is over 2000 times the dose accrued in forming the image of Fig. 4a.

The low dose of our experimental images compared with the 7 GGy tolerable dose implies that scanning Compton X-ray microscopy could achieve higher resolution if the focused spot size was reduced further. For a sample of thickness *L*, the required exposure to achieve a resolution *d* scales as *L⁄d*^4^ (ref. ^7^) whereas the tolerable dose is equal to *d* 10^8^ Gy/nm (ref. ^1^). This means that with the current microscope, it should be possible to form images of 11 nm resolution of the pollen grain at a dose of 1.1 GGy, which could be achieved by appropriately reducing the source size and bandwidth. This scaling assumes that the contrast of the resolved feature stays constant, but as the resolution improves, the contrast of the image improves since density variations are no longer being averaged over. This has the effect of reducing the exponent in the scaling of the required dose with resolution, implying that the achievable resolution could be better than 11 nm.

The contrast and signal to noise of the images obtained here could be further improved, reducing the required exposure and hence the dose, by further reducing the background signal and by increasing the detector solid angle. The background signals for each of the images of Fig. 4 are given in Table 1. The minimum counts per pixel for each image is a significant proportion of the range of counts of that image. This background primarily originates from Compton scattering from the air. Since the density of air is 1.2 × 10^−3^ g/cm^3^, a 1 cm air path contributes as much as 10 µm thickness of typical biological material. Whatever the cause, the background adds to the apparent thickness *L* of the sample and thus adds to the required exposure and dose. By using a helium or vacuum environment it should be possible to approximately half the required dose for a given image signal to noise ratio, which would reduce the damage limited resolution by another factor of 0.5^1/5^ = 0.87. The two dark-field detectors used here collected about 17% of the total Compton scattering signal. A gas-detector concept was recently described to increase this to almost 100%, which would reduce the dose to achieve a particular signal by this factor of 0.17 (ref. ^9^). The tungsten plate to block scattering from upstream components could be replaced by a conical structure, e.g. with a vertex angle of 90°. A gas detector that accepted all photons between scattering angles *θ* of 20° to 135°, for example, would have a coverage of 80%, which would reduce the dose for collecting images of a given signal to noise ratio by a factor of 0.21 (from 0.8 MGy to 0.2 MGy) to enable damage-free imaging of biological objects to about 9 nm resolution. As mentioned above, the required dose is sample dependent and depends on the variation of density in the volume. This is illustrated by the image of the spirulina, which does not show much contrast beyond the cell walls. From the 9 MGy dose of its image, the same scaling predicts a damage-limited resolution of 15 nm for this object.

Adding energy resolution to the detector would give the possibility to display simultaneous fluorescence maps of heavier elements, which are usually present in biological systems in trace amounts. Achieving both high spectral and angular resolution over large solid angles, such as with a pnCCD detector^30^ would enable additional maps of the sample structure to be obtained from the Compton and elastic scattering. For example, the ratio of elastic to inelastic scattering could be used to map the effective atomic number^31^, and diffraction tomography data^32^ could be simultaneously measured by discriminating photons based on their energy.

The images presented here show the potential for low-dose imaging of biological samples. With new storage ring designs that promise large increases in hard X-ray brightness at synchrotron radiation facilities^10,33^, our results imply that scanning Compton X-ray microscopy should be viable for imaging biological and soft-matter samples at resolutions better than 10 nm.

## Materials and methods

### Multilayer Laue lenses

Multilayer Laue lenses, consisting of 7366 bi-layers of SiC and WC were fabricated in our laboratory by masked-layer deposition using magnetron sputtering onto flat silicon substrates^19^. Focusing is achieved by diffraction from layers that vary in period approximately inversely with distance from the optical axis, and which vary in their tilt to ensure the Bragg diffraction condition is satisfied at all positions. The variation in period was programmed by the velocity of the substrate through the sputtering plumes of the two materials during the deposition process and the tilt was achieved by shadowing the substrate with a straight-edge mask. Two lenses, with focal lengths of 15.0 mm and 15.6 mm at 60 keV, and heights of 51 µm and 52 µm, were cut using a Xe-plasma focused ion beam (FIB) from the required positions in the deposited structure along the shading gradient^34^, as confirmed by measurements of the diffraction as a function of lens tilt angle using a laboratory source at a photon energy of 17.5 keV (ref. ^35^). At the position where the lenses were sliced, the bi-layer period varied from 15.0 nm to 4.4 nm. The lens aperture is off axis, with the maximum period located 21 µm from the optical axis. Since each lens focuses in only one direction (like a cylindrical lens), they were mounted orthogonally, spaced apart by 0.6 mm, to focus in both directions to a common plane, with a numerical aperture (NA) of 1.7 × 10^−3^ in both the horizontal and vertical directions. The lenses were both cut to a thickness of 35 µm in the direction of beam propagation to optimise their diffraction efficiencies as estimated by dynamical diffraction calculations^36^. The efficiency was measured at 60 keV as 80% for a single lens, or 65% for the pair.

**Fig. 5:**
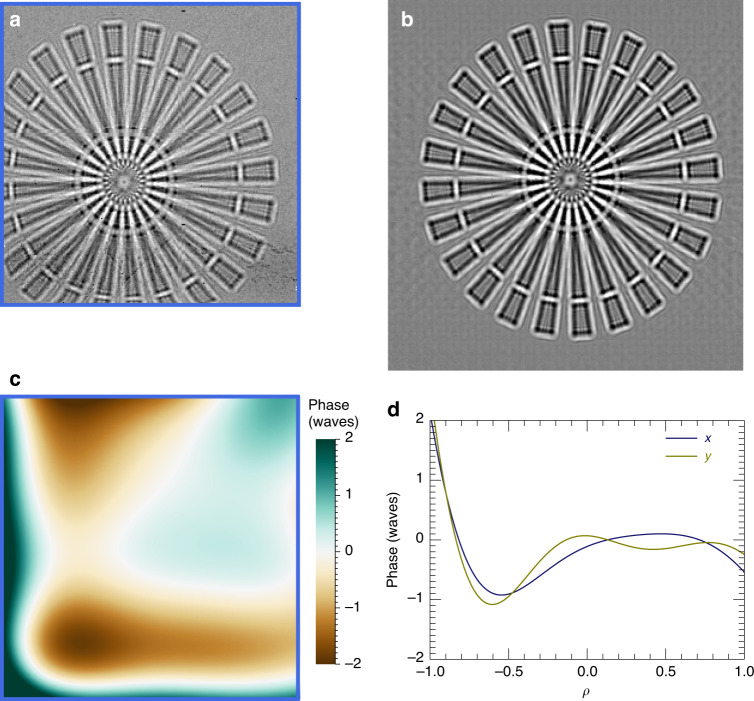
Wavefront sensing at 60 keV. **a** Projection image of the 20-µm diameter gold Siemens star object as recorded on the downstream GaAs detector with an exposure of 500 ms, after dividing by the no-sample signal. Reference image (**b**) and phase map (**c**) obtained by ptychographic speckle tracking processing of a dataset of projection images. The best-fit tilt and defocus has been subtracted from the phase map. The blue frames in (**a**) and (**c**) mark the edge of the pupil. **d** Plots of the phase in the *x* and *y* directions as a function of the normalised pupil coordinate *ρ* after adjusting the lenses to correct for astigmatism. These plots represent the aberrations of the individual lenses. The phase is plotted in units of waves, with a wavelength of 0.02 nm

The lenses were aligned and characterised, in our microscope set-up located in the second experimental hutch at beamline P07 of the PETRA III synchrotron radiation facility, by monitoring the wavefront of the focused beam using the method of ptychographic X-ray speckle tracking (PXST)^25,37^. For this, the same 20-µm diameter gold Siemens star structure imaged in Fig. 3 was placed 6.0 mm downstream of the focus, where the beam size roughly matched the diameter of the object, and projection images of the structure were recorded on the downstream GaAs detector at a magnification of 675. These images had very low contrast with an attenuation of the gold measured to be 0.00602. Given a density of 19.3 g/cm^3^, and a total attenuation cross section of Au at 60 keV of 4.529 cm^2^/g (ref. ^22^), the thickness of the gold is calculated to be 703 nm, consistent with the nominal thickness of 750 nm. At a distance of 4.2 m from the focus, the divergent beam expands to a width of 13.75 mm and illuminates an area of 250 × 250 pixels. A 500 ms exposure, with an incident flux of 1.06 × 10^9^ ph/s is shown in Fig. 5a, after dividing by the no-sample exposure. This projection image is a Gabor hologram of the Siemens star. The visible diffraction fringes, especially inside and around the endcaps of the spokes of the Siemens star, indicate a spatial coherence that is roughly equal in the horizontal and vertical directions. (The degree of coherence may be surprising, given the focus spot size estimated in Fig. 3e, but it should be noted that the focal spot is not an incoherent source—it is an image of one).

The hologram is distorted by the wavefront aberrations of the diverging lens. Quite prominent distortions can be observed along the left and bottom edges of the projection image in Fig. 5a, for example, where the arms of the Siemens star appear to bend. Without knowing the actual structure of the Siemens star, the wavefront distortions can nevertheless be revealed by stepping the object over many different positions in the transverse plane. The trajectory of any particular feature in the Siemens star forms a map of the effect of the wavefront. We rastered the sample in a grid of 21 × 21 points with a step size of 1 µm. From this dataset, the PXST algorithm iteratively finds the undistorted image (Fig. 5b) and the map of those distortions across the wavefield which can then be integrated to give a map of the phase (Fig. 5c). The 2D map can be decomposed into a set of orthogonal functions over the domain of the lens pupil defined by the normalised lens coordinates (*ρ*_*x*_*,ρ*_*y*_) which range from (-1,-1) to ( + 1, +1). The functions are obtained by a Gram-Schmidt orthogonalisation of Zernike polynomials^38^ calculated in the square domain and hence preserve their character. The 0–90° astigmatism term indicates the longitudinal displacement of the foci of the two lenses, which can then be moved further or closer to each other to achieve a common focus. The 45° astigmatism term indicates the non-orthogonality of the lenses, which can then be adjusted by rotating one of the lenses^39^. Once the lenses were made confocal and orthogonal, the RMS wavefront error across the pupil after subtracting piston, tilt, and focus, was equal to 0.80 waves (corresponding to an RMS optical path length error of 0.016 nm). The 2D map is separable into phase profiles of the two individual lenses, plotted in Fig. 5d. The aberration of the two lenses is similar, which is expected since both lenses were cut from the same deposited structure. The coherent point spread function shown in Fig. 3d was obtained from the wavefront map by a Fourier transform^40^.

The off-axis nature of the lens aperture exacerbates the effect of the chromatic aberration inherent to a diffractive lens. The focal length of such a lens is inversely proportional to the wavelength. A change of wavelength will not only cause a defocus, but also a shift of the beam. When integrating over the range of wavelengths supplied to the lens, this shift leads to an asymmetry in the diagonal direction as shown in Fig. 3e.

### Compton scattering geometry

The differential scattering cross section for inelastic scattering, per atom, for an element of atomic number *Z* is given by

4 $$\frac{{\rm{d}}{\check{\sigma }}_{{\rm{inc}}}}{{\rm{d}}\varOmega }={S}_{Z}\left(q\right)\frac{{\rm{d}}{\check{\sigma }}_{{KN}}}{{\rm{d}}\varOmega }$$

where *S*_*Z*_ is the incoherent scattering function of the element, $q=({\rm{sin }}\theta)/(2\lambda )$ is a parameter proportional to the photon momentum transfer for elastic scattering, and $d{\check{\sigma }}_{{KN}}/d\varOmega$ is the polarised Klein-Nishina cross section for an electron:

5 $$\frac{{\rm{d}}{\check{\sigma }}_{{KN}}}{{\rm{d}}\varOmega }=\frac{{r}_{e}^{2}}{2}{p}^{2}\left(p+\frac{1}{p}-2{{\rm{sin }}}^{2}\theta {{\rm{cos}}}^{2}\phi \right)$$

where *r*_*e*_ is the classical radius of the electron, *p* is the ratio of scattered to incident photon energies (Eq. 3), and *ϕ* the azimuthal angle of the scattered photon^41^. Similarly, the elastic scattering cross section for an atom is related to the Thompson scattering cross section through the atomic scattering factor *F*_*Z*_ as

6 $$\frac{{\rm{d}}{\check{\sigma }}_{{\rm{coh}}}}{{\rm{d}}\varOmega }={F}_{Z}^{2}\left(q\right)\frac{{\rm{d}}{\check{\sigma }}_{T}}{{\rm{d}}\varOmega }$$

7 $$\frac{{\rm{d}}{\check{\sigma }}_{T}}{{\rm{d}}\varOmega }=\frac{{r}_{e}^{2}}{2}\left(1-{{\rm{sin }}}^{2}\theta {{\rm{cos }}}^{2}\phi \right)$$

The cross section per unit mass of a particular element for either interaction is then found as

8 $$\frac{{\rm{d}}\sigma }{{\rm{d}}\Omega }=\frac{{N}_{A}}{{A}_{Z}}\frac{d\check{\sigma }}{d\Omega }$$

for the atomic mass *A*_*Z*_ and Avogadro’s constant *N*_*A*_. Experimental and theoretical estimates of the functions *S*_*Z*_(*q*) and *F*_*Z*_*q* are tabulated in several places. We used the estimates of Hubbell^21^ to ensure consistency between the elastic and inelastic scattering factors. In the forward direction, *S*_*z*_(0) → 0 and ${F}_{z}^{2}\left(0\right)\to {Z}^{2}$, whereas at high angles and high photon energies *S*_*z*_(*q*) → *Z* and ${F}_{z}^{2}\left(q\right)\to 0$. Plots of d*σ*_inc_/dΩ are shown in Fig. 1c, d.

The expected signal on each of the dark-field detectors can be computed by integrating the differential cross section over the solid angle of each detector pixel. We consider a local coordinate system corresponding to a detector a distance *L* from the origin (at the sample) with a pixel located at (*x*_*d*_*, y*_*d*_*, L*) and the local *z* axis passing through the center of the detector panel. These local coordinates coincide with the global coordinate system (indicated in Fig. 1a) if the detector was placed a distance *L* downstream of the sample with its face normal to the beam axis. Compared with that position, the detector was rotated in the *y-*z plane about the origin by an angle *α*. The distance of the pixel to the origin remains equal to

9 $$\left|{\boldsymbol{r}}\right|=\sqrt{{x}_{d}^{2}+{y}_{d}^{2}+{L}^{2}}$$

and thus the solid angle subtended by the pixel from the origin is

10 $${\varOmega }_{p}=\frac{{A}_{p}}{{\rm{|}}{\boldsymbol{r}}{{\rm{|}}}^{2}}\hat{{\boldsymbol{r}}}\cdot \hat{{\boldsymbol{n}}}=\frac{{A}_{p}L}{{\left({x}_{d}^{2}+{y}_{d}^{2}+{L}^{2}\right)}^{3/2}}$$

where $\hat{{\boldsymbol{n}}}$ is the unit normal of the detector face and *A*_*p*_ is the area of a pixel (55 µm x 55 µm in our case). The global coordinates of the pixel of the rotated detector are

11 $${\boldsymbol{r}}=\left({x}_{d},{y}_{d}\,{\rm{cos}}\,\alpha +L\,{\rm{sin}}\,\alpha ,-{y}_{d}\,{\rm{sin}}\,\alpha +L\,{\rm{cos}}\,\alpha \right)$$

The unit vector in the direction of the pixel, in terms of the scattering angles *θ* and *ϕ* is

12 $$\hat{{\boldsymbol{r}}}=\frac{{\boldsymbol{r}}}{\left|{\boldsymbol{r}}\right|}=\left({\rm{sin }}\,\theta \,{\rm{cos }}\,\phi ,{\rm{sin }}\,\theta \,{\rm{sin }}\,\phi ,{\rm{cos }}\,\theta \right)$$

Thus,

13 $${\rm{cos }}\,\theta =\frac{-{y}_{d}\,{\rm{sin }}\,\alpha +L\,{\rm{cos }}\,\alpha }{\sqrt{{x}_{d}^{2}+{y}_{d}^{2}+{L}^{2}}}$$

14 $${\rm{tan}}\,\phi =\frac{{y}_{d}\,{\rm{cos }}\,\alpha +L\,{\rm{sin }}\,\alpha }{{x}_{d}}$$

The detector signal at a pixel (*x*_*d*_*,* *y*_*d*_) and detector distance *L* is then computed using the above expressions to form

15 $${\sigma }_{p}\left({x}_{d},{y}_{d},L,\alpha \right)=\frac{{\rm{d}}\sigma }{{\rm{d}}\varOmega }\left({x}_{d},{y}_{d},L,\alpha \right){\varOmega }_{p}\left({x}_{d},{y}_{d},L\right)$$

The total detector cross section *σ*^det^ is computed by summing this expression over all active and unshadowed pixels of the detector. For the upper detector, *L* = 31 mm and *α* = 46°, while for the lower detector, *L* = 60 mm and *α* = 19°. With these parameters, the incoherent and coherent detector cross sections for nitrogen (and hence for organic material) are 0.023 cm^2^/g and 0.001 cm^2^/g, respectively for the upper detector, and 0.010 cm^2^/g and 0.001 cm^2^/g for the lower detector.

**Fig. 6:**
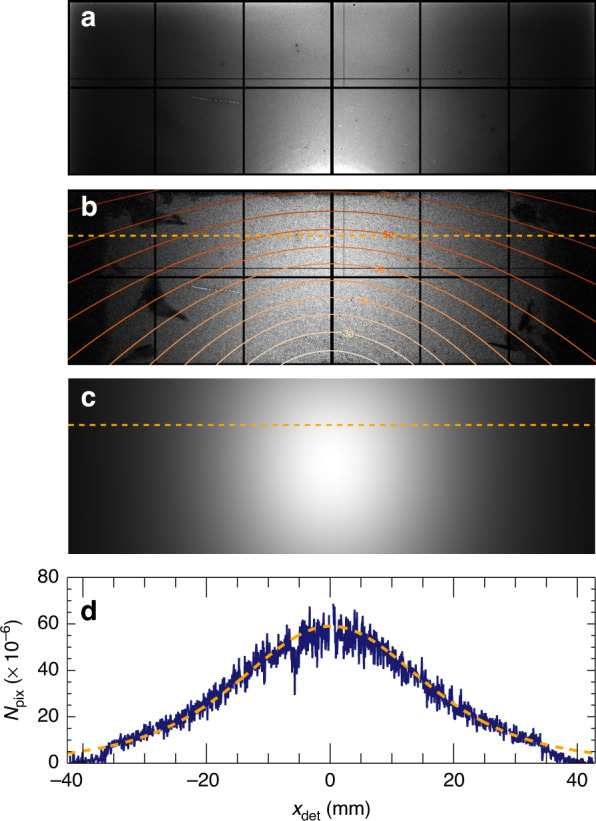
Compton scattering profile. **a** Detector signal in the upper detector without a sample in place, with counts ranging from 0 (black) to 2 × 10^−4^ counts/pixel/ms (white). **b** Detector signal from the rim of the silicate diatom sample shown in Fig. 4c, after subtracting the no-sample map, with counts ranging from 0 (black) to 8 × 10^−5^ counts/pixel/ms (white). Contours of constant *θ* are shown. **c** Computed detector signal, scaled to the same maximum counts as (**b**). **d** Line out of the detector signal along the orange dashed lines of (**b**) and (**c**)

A map of the calculated Compton scattering signal of a silicate sample (with assumed stoichiometry of SiO_2_) is displayed in Fig. 6c, in comparison with the no-sample subtracted measurement on the upper dark-field detector from the diatom sample, shown in Fig. 6b. The calculation matches the distribution across the entire detector, except for shadows cast by the pyramid-shaped shielding structure. This can also be compared in the plot of the signal along a particular row of the detector, shown in Fig. 6d. Unlike the example of the Si wedge given in Fig. 2, the actual sample mass is unknown, but the calibration of the detector cross section for Si allows us to place the dark-field images of Fig. 4 onto an absolute scale of projected density.
